# Neurotrophins and their receptors in the peripheral nervous system and non-nervous tissue of fish

**DOI:** 10.1007/s10695-025-01453-7

**Published:** 2025-01-31

**Authors:** Aldo Isaac Carrillo-Muñoz, Sharet Y. R-Jaimes, Guadalupe C. Hernández-Hernández, Francisco Castelán

**Affiliations:** 1 Centro Tlaxcala de Biología de La Conducta, Universidad Autónoma de Tlaxcala, 90070 Tlaxcala, Mexico; 2 Facultad de Ciencias de La Salud, Universidad Autónoma de Tlaxcala, 90750 Zacatelco, Mexico; 3 Doctorado en Ciencias Biológicas, CTBC, Universidad Autónoma de Tlaxcala, 90070 Tlaxcala, Mexico; 4 Departamento de Biología Celular y Fisiología, Instituto de Investigaciones Biomédicas, Universidad Nacional Autónoma de México, 90070 Tlaxcala, Mexico

**Keywords:** Teleost, Trophic factors, Non-neural tissue, Vertebrate molecular evolution, Animal model

## Abstract

Trophic factors, such as neurotrophins, are fundamental for cellular processes including differentiation, growth, survival, and regeneration. These molecules exhibit significant morphological and phylogenetic conservation throughout the animal kingdom, indicating conserved functions. In fish, the oldest and most diverse group of vertebrates, neurotrophins, and their receptors play pivotal roles not only within the central nervous system but also in various peripheral tissues. They are distributed in mechanosensory, muscle, skin, respiratory, circulatory, digestive, endocrine, urinary, reproductive, and immune systems, suggesting their involvement in the development and maintenance of all tissues/organs/systems. Despite this broad distribution, studies focusing on these molecules outside of the central nervous system have been limited to just 12 fish species. These investigations have revealed diverse expression patterns across different ages and tissues/organs/systems, expanding our comprehension of their functions beyond the central and peripheral nervous systems. Notably, BDNF and NT-3 are prominently expressed outside the central nervous system, particularly in mechanosensory and digestive tissues, whereas NGF is predominantly observed in mechanosensory and urinary systems. The expression and localization of neurotrophins and their receptors vary among organs, underscoring tissue-specific roles. Further research is imperative to decipher the precise functions and mechanisms of action of neurotrophins and their receptors in diverse fish tissues. Enhanced efforts are needed to include a broader range of fish species in these studies to advance our understanding of these agents in complex vertebrates, thereby shedding light on tissue development, regeneration, and maintenance, with potential implications for addressing organ-related issues.

**Graphical Abstract:**

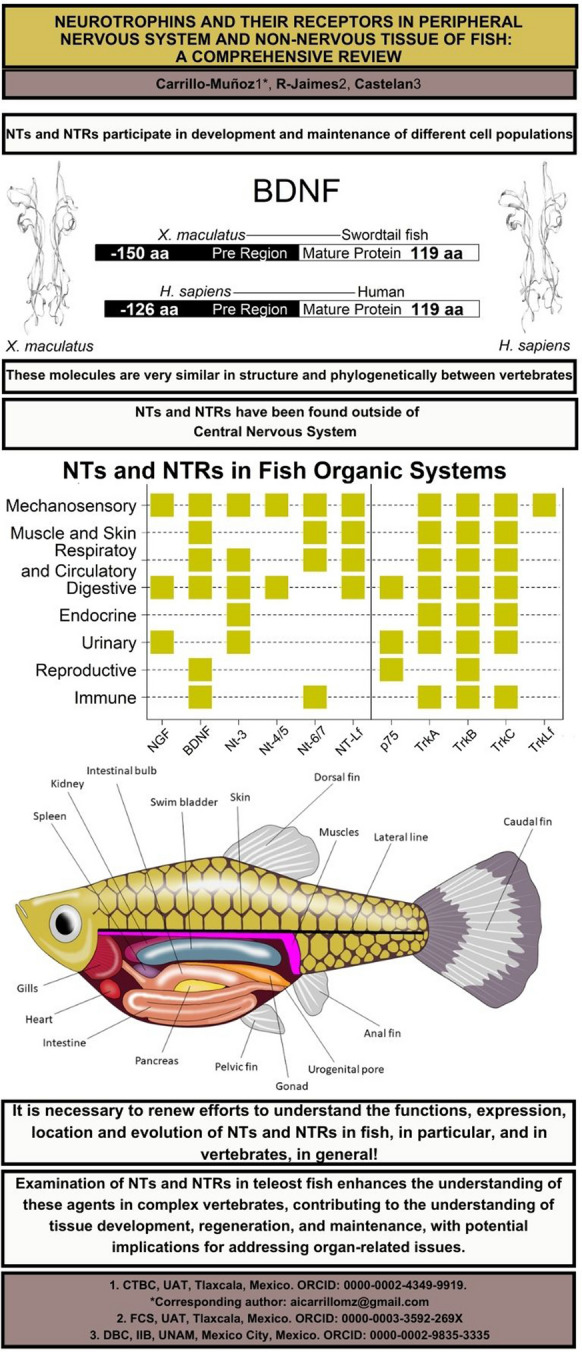

## Introduction

Trophic factors, as cellular mediators, assume various functions in the differentiation, migration, growth, survival, regeneration, and death of distinct cell populations. They facilitate essential communication between systems across all developmental stages of vertebrates, from embryos to advanced developmental stages (Ramón y Cajal 1928; Levi-Montalcini 1987; Götz and Schartl 1994; Ibáñez 1995; Tessarollo 1998; van Kesteren et al. 1998; Fariñas 1999). Neurotrophins (NTs) are a class of trophic factors that engage with their receptors (NTRs) and trigger biological responses, predominantly with the central (CNS) and peripheral nervous system (PNS) (Bijlsma et al. 1984; Barde 1989; Korsching 1993; Barbacid 1995; Davies and Wright 1995; Fariñas and Reichardt 1996). Nevertheless, NTs also drive cellular processes in non-neural tissues (NNT) (Levi-Montalcini 1987; Lomen-Hoerth and Shooter 1995; Otten and Gadient 1995; Yamamoto et al. 1996; Tessarollo 1998; Aloe et al. 1999; Hannestad et al. 2000; Aloe 2001; Sariola 2001), such as intestinal and epithelial endocrine cells (Esteban et al. 1995; Shibayama and Koizumi 1996; Lucini et al. 1999; Domeneghini et al. 2001), pancreas (Kanaka-Gantenbein et al. 1995; Miralles et al. 1998), and immune system (Ehrhard 1993a, b; Vega et al. 2003; Calabrese et al. 2014; Minnone et al. 2017), among others (see Yamamoto et al. 1996; Sariola 2001). Notably, NTs and NTRs show distinct expression in cells and tissues, often showcasing overlapping or divergent functions across vertebrates (Nittoli et al. 2018).

Phylogeny reconstruction has demonstrated a high degree of conservation of NTs and NTRs among vertebrates (Götz et al. 1994; Hallböök et al. 1991, 1998; van Kesteren et al. 1998). In fish species, these trophic factors have been identified in neural and NNTs (Martin et al. 1995; Lai et al. 1998; Nilsson et al. 1998; Lucini et al. 1999; de Girolamo et al. 1999, 2000; Hannestad et al. 2000; Heinrich and Lum 2000; de Girolamo and D'Angelo 2021).

Teleost fish, also known as bony fish, share many common structures and biological functions with mammals, like humans (Teame et al. 2019). Bony fish are excellent animal models for developmental biology, genetics, comparative anatomy, and physiology (Powers 1989; Matsui 2017; Heinrich and Lum 2000; Teame et al. 2019; Bera et al. 2020; de Girolamo and D´Angelo 2021). The zebrafish (*Danio rerio*) has provided compelling insight into the roles of NTs and NTRs in both early development and adulthood. Furthermore, fish exhibit a remarkable ability to regenerate various tissues and neurons throughout their lifespans. This attribute not only aids in understanding their function in fish physiology but also provides valuable insights applicable to all other groups of vertebrates, both amniotes and anamniotes, which share development and nerve features (Northcutt and Gans 1983; Moreno and González 2006, 2007; Heinrich and Lumm 2012; Zhang et al. 2012), and differ in genome organization, physiology, and reproductive strategies (Olmo et al. 1989; Meriri et al., 2021).

A recent comprehensive review has explored the involvement of NTs and NTRs specifically within neural tissues of bony fishes (Girolamo and D’Angelo 2021). However, the scope of the data within this review is limited to observations in the zebrafish (*D. rerio*), two killifish species (*Nothobranchius guentheri* and* N. furzeri*), and two swordtail fish species (*Xiphophorus maculatus* and *X. helleri*). Since NTs and NTRs are expressed in various tissues outside the CNS, we expected a similar pattern in fish. However, our analysis revealed a significant gap in the research on this topic. Only a few studies have explored their expression and localization in different tissues/organs/systems. This is why the current review aims to examine the expression patterns of various NTs and NTRs beyond the brain, encompassing non-neural tissues (NNT) in teleost fish.

Firstly, this review focuses on the mechanosensory system, which includes the inner ear and the lateral line in teleost fish (Cernuda-Cernuda and García-Fernández 1996). Although these structures receive direct nerve connections from the cranial nerves, the lateral line extends across the entire body (Davis and Northcutt 1983; Kotrschal et al. 1998). Secondly, the review delves into the expression and localization of NTs and NTRs in diverse systems such as muscles, skin, respiratory and circulatory systems, digestive tract, endocrine glands, urinary and reproductive systems, and finally, the immune system.

In compiling this review, we searched for publications on the PubMed and Google Scholar platforms. Several key words and combinations of them were used (neurotrophins fish, neurotrophic factor fish, neurotrophins receptors fish, teleost neurotrophins, teleost neurotrophins receptors, BDNF, brain-derived neurotrophic factor, NGF, nerve growth factor, NT-3, NT-4/5, NT-6/7, NT-Lf, tyrosine kinase receptor, TrkA, TrkB, TrkC, and p75ntr). The collected research studies focus on the presence and distribution of various neurotrophic factors, including nerve growth factor (NGF), brain-derived neurotrophic factor (BDNF), neurotrophin 3 (NT-3), neurotrophin 4 and 5 (NT-4/5), neurotrophin 6 and 7 (NT-6/7), and a neurotrophin discovered in *Lampetra fluviatilis* (NT-Lf). Additionally, we examined neurotrophin receptors (NTRs), encompassing low-affinity receptors (p75NTR) and high-affinity receptors (TrkA, TrkB, TrkC, Lf-Trk1, and Lf-Trk2), particularly those related to the lateral line and non-neural tissues (NNT). Of the articles found, only those that considered the expression and localization of NTs and NTRs genes or proteins in tissues and organs outside the central nervous system were selected. Besides, we also considered the articles that analyzed the lateral line and the inner ear distributed throughout the entire body of the fish. Additionally, we integrated jawless fish that showed expression of these molecules outside the CNS.

**Fig. 1:**
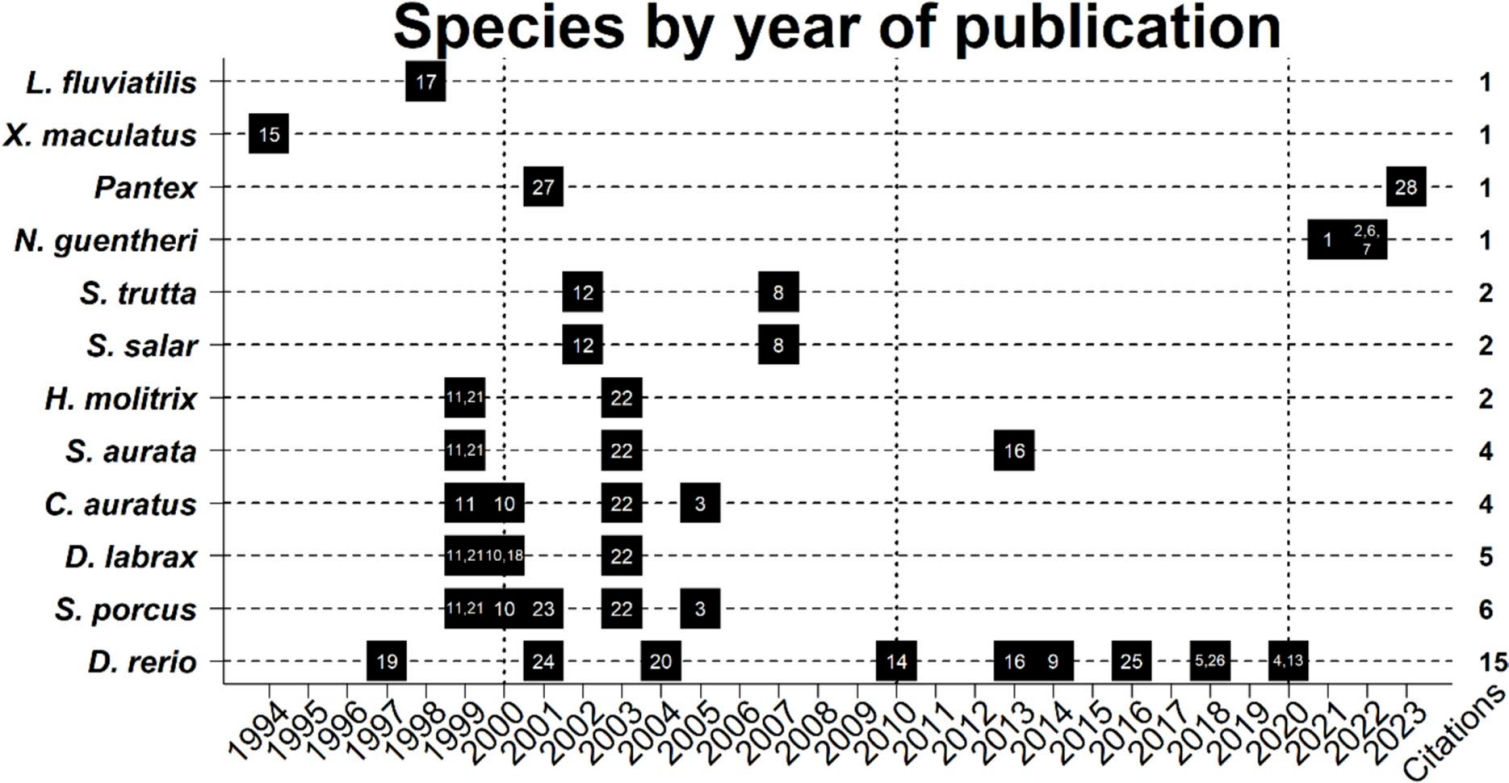
Fish species showing expression of neurotrophins (NTs) or their receptors (NTRs) by year of publication. Each black box represents the publication(s) for a species in a given year, the numbers inside the boxes represent the corresponding citation(s): (1) Aragona et al. (2021); (2) Aragona et al. (2022); (3) Arcamone et al. (2005); (4) Blanco et al. (2020); (5) Cacialli et al. (2018); (6) Cacialli (2022); (7) Cacialli and Lucini (2022); (8) Catania et al. (2007); (9) De Felice et al. (2014); (10) de Girolamo et al. (2000); (11) de Girolamo et al. (1999); (12) Germanà et al. (2002); (13) Germanà et al. (2020); (14) Germanà et al. (2010); (15) Götz et al. (1994); (16) Guerra et al. (2013); (17) Hallböök et al. (1998); (18) Hannestad et al. (2000); (19) Hashimoto and Heinrich (1997); (20) Heinrich and Pagtakhan (2004); (21) Lucini et al. (1999); (22) Lucini et al. (2003); (23) Lucini et al. (2001); (24) Lum et al. (2001); (25) Montalbano et al. (2016); (26) Nittoli et al. (2018); (27) Radaelli et al. (2001); (28) Tuz-Sasik et al. (2023). See “References” for full citations. The figure was created using R software (ver. 4.2.2 “Innocent and Trusting” R Core Team 2022)

Our analysis revealed that only 12 fish species, including a jawless fish species (*L. fluviatilis*, situated at an earlier evolutionary stage among fishes (Hallböök et al. 1998), have been investigated regarding NTs and/or NTRs in NNT. The limited exploration of species highlights the necessity for additional studies to enhance our comprehension of the roles, expression, distribution, and evolutionary aspects of NTs and NTRs among diverse vertebrates. Notably, the number of publications investigating NTs and NTRs in fish NNT exhibited a decline over the initial decade of this century, with a further decrease continuing into the subsequent decade (Fig. 1).

### Neurotrophins (NTs) and their receptors (NTRs)

The first NTs documented in tetrapods were NGF, BDNF, NT-3, and NT-4/5 (Hallböök et al. 1991). Additionally, neurotrophin-6 and neurotrophin-7 (NT-6/7), along with a neurotrophin specific to lampreys (NT-Lf), were exclusively identified in fish (Götz et al. 1994; Hallböök et al. 1998; Lai et al. 1998; Nilsson et al. 1998).

**Fig. 2:**
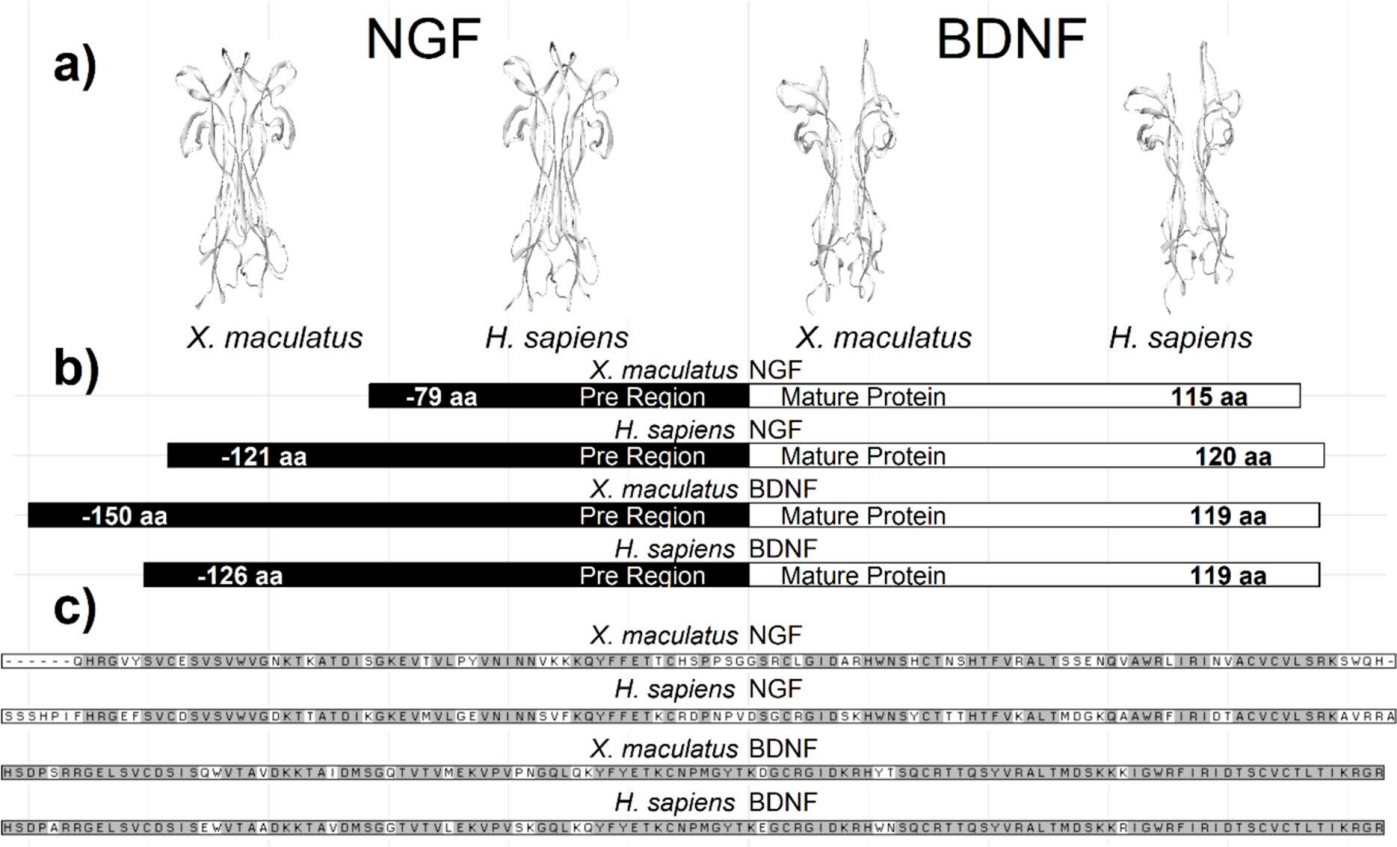
Vertebrate NTs.** a** Three-dimensional structures of homodimer of mature NT. Nerve growth factor (NGF) and brain-derived nerve factor (BDNF) of fish and human 3D structures are represented (3D images were obtained with Swiss-Model Viewer (Guex et al. 2009); using NCBI database: NGF *X. maculatus*: CAA42566, *Homo sapiens*: AAH32517; BDNF *X. maculatus*: XP_023187551, *H. sapiens*: CAA62632. **b** Amino acid length of each NT monomers. Pre-region and mature protein are represented; the length of each region in each protein is indicated in black or white, respectively. **c** Amino acid alignment of each mature NT, dashes were inserted to maintain proper alignment, conserved residues are shaded, BLAST analyses indicate that they are homologous sequences (NGF:Fish-Human > 0.02, BDNF:Fish-Human > 0.02) and had similar structure and function (NGF:Fish-Human = 66.970% identity, BDNF:Fish-Human = 90% identity) (see Götz & Schartl 1994). The figure was created using R software (ver. 4.2.2 “Innocent and Trusting” R Core Team 2022)

The mature NTs are small and homodimeric proteins formed by two monomers comprising roughly 120 amino acid residues (Davies 1994; Götz and Scharlt 1994; Ibáñez 1995; Lai et al. 1998). Neurotrophin family members identified across vertebrates exhibit comparable primary and three-dimensional structures; these structures feature conserved regions with over 50% similarity in their amino acid sequence (Fig. 2). NTs contain six cysteine residues, and the amino acids at the end of the pre-region play a crucial role for proteolytic anchoring during its growth and maturation. Furthermore, each of the NTs encompasses five regions characterized by specific motifs (Hallböök et al. 1991; Lewin and Barde 1996; Lai et al. 1998; Fariñas 1999; Heinrich and Lum 2000).

NTs and NTRs are evolutionary conserved molecules between vertebrates, showing amino acid and nucleotides similarities from fish to mammals (Hallböök et al. 1991; Shelton et al. 1995; van Kesteren et al. 1998; Hallböök 1999; Heinrich and Lum 2000). The discovery of homologous NTs and NTRs in different lineages points to a shared ancestral origin during early vertebrate evolution and suggests gene duplication near the root of vertebrate evolution (Hallböök et al. 1991; Götz and Schartl 1994; Holland et al. 1994). Phylogenetic studies have demonstrated that this gene duplication event occurred in the early evolution of vertebrates, giving rise to all known NTs and NTRs across different vertebrate lineages (Hallböök 1999; Tettamanti et al. 2010). Nevertheless, it should be noted that the NT-NTR system is not a vertebrate innovation, as analogous molecules also exist in invertebrates (Hallböök et al. 2006; Zhu et al. 2008; Tettamanti et al. 2010).

There are two types of receptors for NTs distinguished by their affinity, either low or high (Meakin and Shooter 1992; Bothwell 1995), which mediate biological actions through the phosphorylation and activation of different membrane receptors (Lewin and Barde 1996; Lai et al. 1998; Fariñas 1999; Hallböök 1999). The low-affinity receptor, termed the p75 neurotrophin receptor (p75NTR), binds to all immature NTs peptides in a similar way, regulating processes such as axonal growth, cell cycle, and synaptic plasticity (Bothwell 1996; Lee et al. 2001; Ibáñez and Simi 2012). On the other hand, high-affinity receptors belong to the transmembrane proteins of the tyrosine-kinase family (Tropomyosin-related kinase, TrkA, B, C). They consist of an extracellular domain characterized by a cysteine-rich cluster, followed by the leucine-rich repeat domain in a triplet, a cysteine-rich domain, and two similar immunoglobulin domains. Each of these receptors spans the membrane once and ends with a cytoplasmic domain consisting of a tyrosine kinase domain surrounded by several tyrosines that serve as phosphorylation-dependent docking sites for cytoplasmic adapters and enzymes, allowing signal transduction (Vega et al. 2003; Reichardt 2014).

Overall, the Trk receptor affinities for each NT can be summarized as TrkA:NGF, TrkB:BDNF, and TrkC:NT-3 (Barbacid 1993; Ip and Yancopoulos 1996; Lewin and Barde 1996; Reichardt 2006), each exhibiting specific features.

NGF was isolated from snake venom in the 1950s (Levi-Montalcini et al. 1968; Cohen et al. 1956; Thoenen and Barde 1980; Davies and Lindsay 1985; Thoenen et al. 1987; Whittemore and Seiger 1987; Hoehnher et al. 1996). It promotes the survival of Purkinje cells and has been detected in the enteric nervous system of all vertebrate groups (Cohen-Cory et al. 1991), stimulates B-cells of the immune system, and aids in the development of lymphoid organs (Otten et al. 1989; Kannan et al. 1994,1996; Vega et al. 2003). NGF is also expressed in mammalian spermatocytes and spermatids (Olson et al. 1987; Ayer-LeLievre et al. 1988). In fish, the action has been observed in the brain and spinal axons (Weis 1968; Benowitz and Greene 1979; D´Angelo et al. 2014a) and NNT (Lucini et al. 2003; Arcamone et al. 2005).

BDNF, isolated from the brain of pigs, supports peripheral sympathetic and neural crest-derived sensory neurons, as well as cholinergic neurons of the basal forebrain. BDNF also supports sensory neurons derived from placodes and neural crests, along with cholinergic neurons of the basal forebrain and motor neurons (Levi-Montalcini et al. 1968; Hallböök et al. 1991; Sendtner et al. 1992; Barbacid 1993; Hoehnher et al. 1996). In fish, it has been found in the CNS, PNS, and NNT (Caminos et al. 1999; Lum et al. 2001; Heinrich and Pagtakhan 2004; Catania et al. 2007; Vissio et al. 2008; Germanà et al. 2010,2020; Guerra et al. 2013; Sánchez-Ramos et al. 2013; D’Angelo et al. 2014b; Montalbano et al. 2016; Cacialli et al. 2018; Nittoli et al. 2018; Blanco et al. 2020).

NT-3, in addition to coupling with TrkC, interacts with TrkA and TrkB with lower affinity. It was discovered in mammals using homology cloning (Rodríguez-Tébar et al. 1989; Ernfors et al. 1990; Hohn et al. 1990; Kaisho et al. 1990; Maisonpierre et al. 1990; Rosenthal et al. 1990; Segal et al. 1992). NT-3 supports motoneurons, neural crest sensory, locus coeruleus, granular cerebellar, dopaminergic neurons of the substantia nigra, and retinal ganglion cells (Hyman et al. 1991; Friedman et al. 1993; Koliatsos et al. 1993). In fish, it has also been found in CNS, PNS, and NNT (Lucini et al. 2003; Arcamone et al. 2005; Catania et al. 2007; Nittoli et al. 2018).

NT-4 binds to TrkB with high affinity and to TrkA with lower affinity and was discovered in the ovary of frogs via cloning and later in mammals (Berkemeier et al. 1991; Hallböök et al. 1991; Ip et al. 1992; Ibáñez et al. 1993; Hallböök 1999). It acts on motoneurons, sensory neurons of the neural crest, placodes, basal forebrain, and locus coeruleus (Henderson et al. 1993). In fish, it has been observed in and outside of the brain (D’Angelo et al. 2016a; Nittoli et al. 2018).

NT-6 was discovered in swordtail fish (*Xiphophorus*) and NT-7 was found in zebrafish (*D. rerio*) exhibiting morphological and physiological similarities to mammalian NGF and binding to TrkA (Lai et al. 1998; Nilsson et al. 1998; Leggieri et al. 2019). These NT result from an ancestral duplication of vertebrate genes, which also give rise to NGF and NT-3 on one side and BDNF and NT-4/5 on the other (Götz et al. 1994; Hallböök 1999; Hallböök et al. 2006; Zhu et al. 2008; Tettamanti et al. 2010), promoting the survival of sympathetic and sensory neurons from the dorsal root ganglion (Nilsson et al. 1998; Leggieri et al. 2019).

Finally, NT-Lf was discovered in lampreys (*L. fluviatilis*) and binds to their specific receptors TrkLf1 and Trk Lf2 (Hallböök et al. 1998). The early evolution of NTs and NTRs genes is not well understood. Multiple gene copies may have facilitated the subsequent evolution of specialized functions. Hallböök et al. (1998) and Hallböök (1999) suggest that NTs and NTRs genes originated from duplications of ancestral genes in the early vertebrate lineage, giving rise to the modern vertebrate genes. The NTs genes in the ancestors of extant jawless fish likely represent precursors to the neurotrophins of higher vertebrates (NGF, BDNF, NT-3, and NT-4/5). Moreover, Lf-Trk1 and Lf-Trk2 are proposed to be orthologs of the ancestral TrkA/C and TrkB genes, respectively.

## Expression of neurotrophins (NTs) and their receptors (NTRs) in fish

**Fig. 3:**
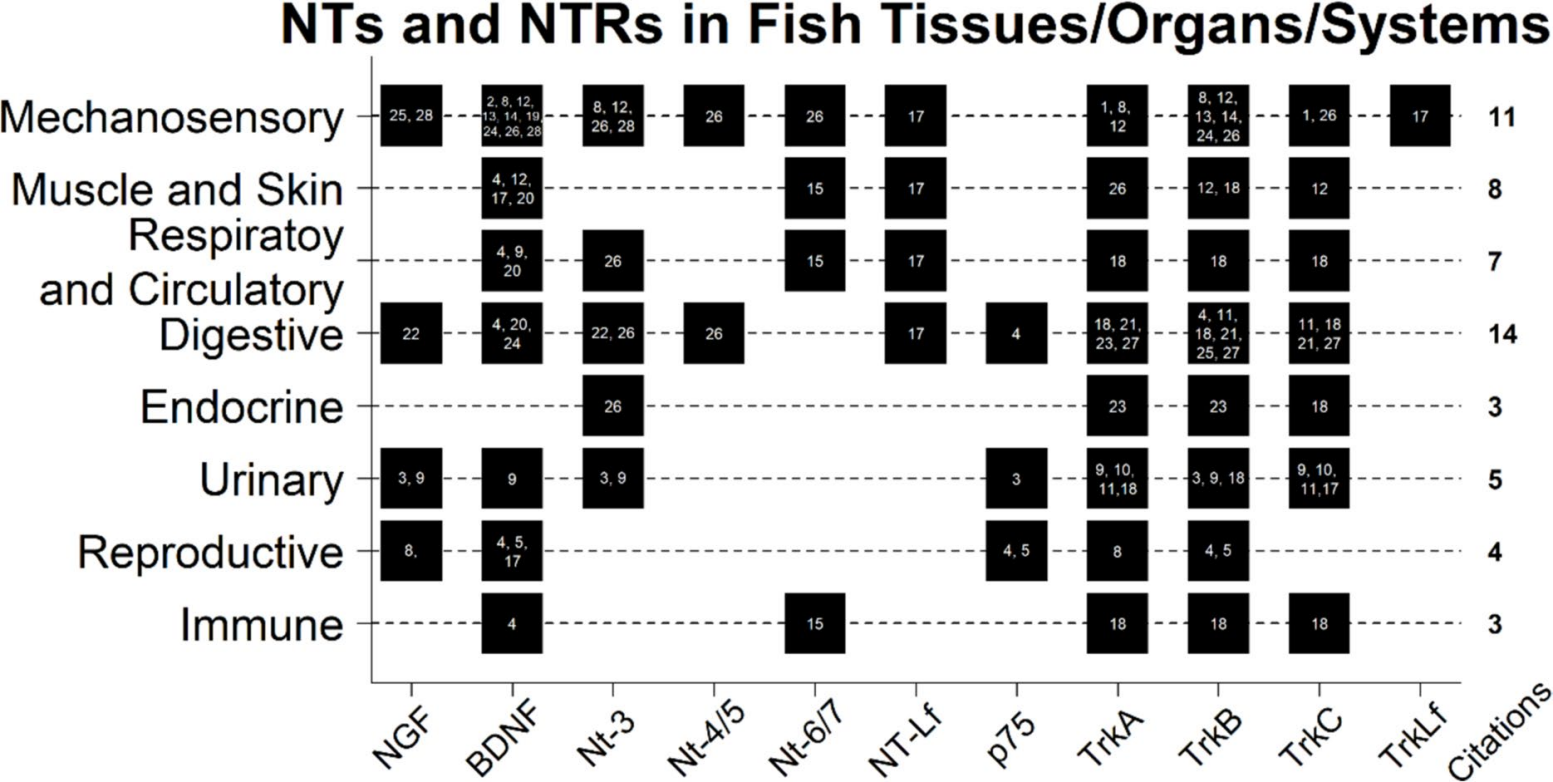
Neurotrophins (NTs) and their receptors (NTRs) outside of CNS and NNT. Each number inside the black boxes represents the citation where it appears. See Fig. 1 to match the number of citations. The figure was created using R software (ver. 4.2.2 “Innocent and Trusting” R Core Team 2022)

**Fig. 4:**
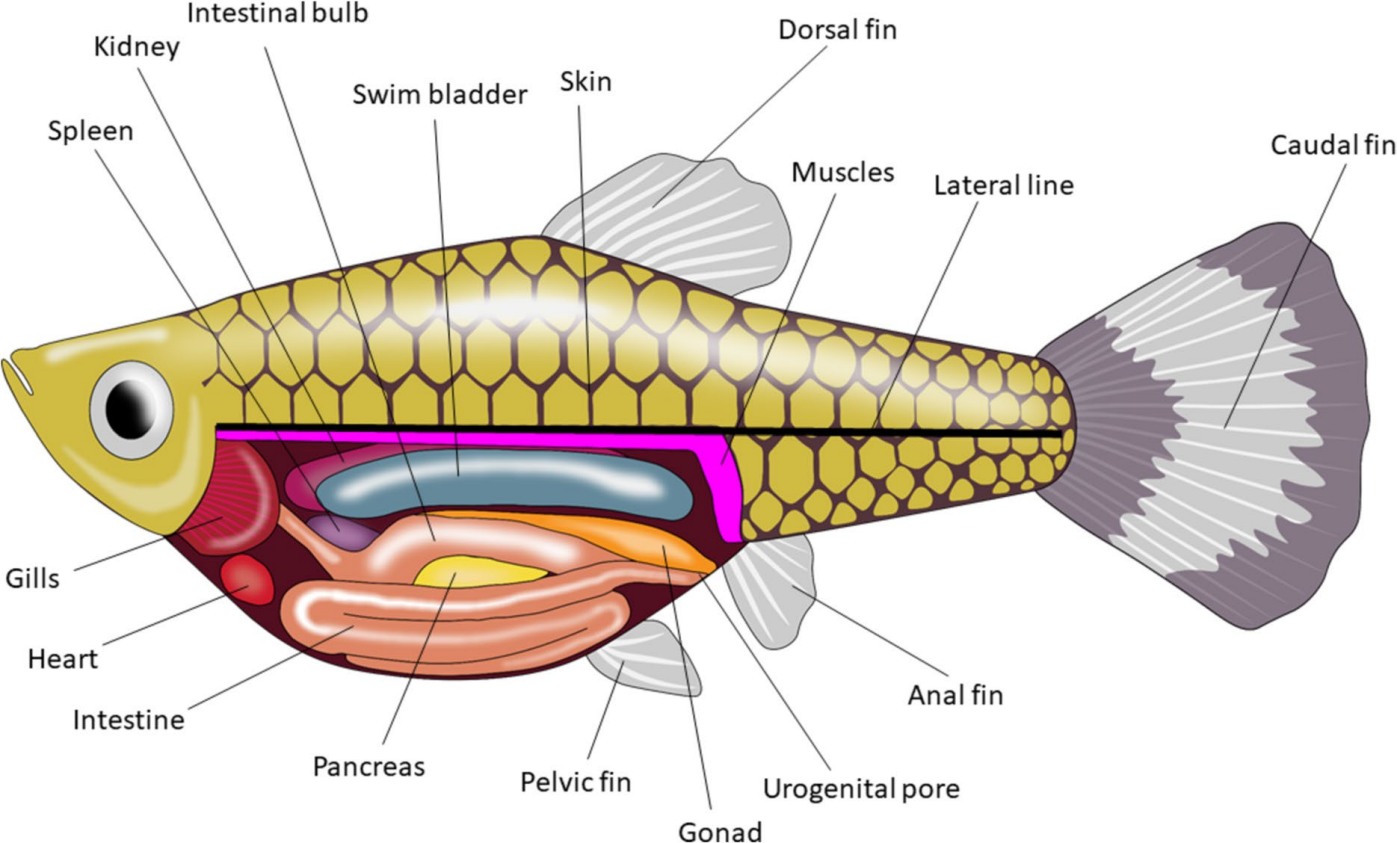
Female guppy fish scheme. Main tissues accounting for either NTs or NTRs are indicated. The figure was created using Adobe Illustrator (ver.27.5)

NTs and NTRs have been reported for tissues/organs/systems beyond the neural ones. Figure 3 provides a general overview of these systems, associating them with the number of publications documenting the presence of NTs or NTRs. Additionally, Fig. 4 depicts a schema illustrating the anatomical regions within fish where these molecules have been detected.

## Mechanosensory system

The mechanosensory system of teleost fish comprises both the inner ear and lateral line system, resembling the sensory system of inner ear found in other vertebrates (Cernuda-Cernuda and García-Fernández 1996; Alexandre and Ghysen 1999; Brignull et al. 2009). This system is under the regulation of NTs, which exert control over various aspects of cell populations, including neuronal proliferation, sympathetic plasticity, development, survival, growth, and differentiation (Henderson 1996; Pirvola et al. 1997; Don et al. 1997; Montcouquiol et al. 1998).

The fish’s inner ear consists of three semicircular canals and membranous sacs (the utricle, the saccule, and the lagena). Each canal has a layer of sensory epithelium or *crista ampullaris* covered by a gelatinous dome, and each sac also has a layer of sensory epithelium (Bang et al. 2001; Catania et al. 2007; Monroe et al. 2015). Meanwhile, the lateral line is comprised of functional organs called neuromasts, composed of hair cells enclosed by support cells, and a neuromast canal that is partially derived from the neural crest. The base of the sensory cells makes synapse with sensory neurons of at least three cranial nerve ganglia. This organ is found on both sides of the head and extends throughout the entire body of teleost fish, functioning by translating water movements into neurochemical signals (Webb 1989; Rouse and Pickles 1991; Collazo et al. 1994; Alexandre and Ghysen 1999; Kasumyan 2003; D'Angelo et al. 2016b; Philip et al. 2024). The neuromasts are distributed in the cephalic and trunk internal canals and on the body surface, forming the pit organs, and each one of them is innervated by a branch of the lateral line nerve (Coombs et al. 1989; Wada et al. 2014).

Hashimoto and Heinrich (1997) were the first to document the expression of BDNF in the developing neuromast of zebrafish (*D. rerio*). Subsequently, BDNF and TrkB were detected in different areas of this fish, including the skin, neuromast, olfactory epithelium, taste organs, cloaca, pectoral fins, and gill arches. The expression of these molecules appears to be restricted as organisms mature from the embryo to 4 days post-fertilization, with a higher density of BDNF transcript copies in the early stages that gradually diminish to levels akin to levels to specialized cells such as ciliary cells of the neuromast, olfactory epithelium, vascular epithelial cells, and taste organelles (Lum et al. 2001). A decrease in immunoreactivity (IR) anti-BDNF and -TrkB has been reported to occur between days 30 and 50 after fertilization for all sensory cells. By adulthood (180 days after fertilization), their expression becomes restricted to a few cells located in the upper periphery of the neuromast (Aragona et al 2022; Germanà et al. 2010). It seems that TrkB-expressing cells in the neuromast depend on BDNF during maturation but become independent towards the end of their development. The potential of BDNF in the regeneration of these cells may be contributing to BDNF/TrkB signaling in the development, maintenance, and regeneration of hair cells (Germanà et al. 2010,2020; Nittoli et al. 2018; Tuz-Sasik et al. 2023).

In addition, transcripts of the NGF, NT-6/7, and TrkC have been identified for the otic vesicle, and NGF, NT-3, and NT-4/5 in the lateral line during early development (Nittoli et al. 2018; Tuz-Sasik et al. 2023). On the contrary Tuz-Sasik et al. (2003) did not find NT-4/5 or NT-6/7 expression in any of the components of the neuromast.

In fingerlings from two salmon species (*Salmo salar* and *S. trutta*), IR anti-NTs and -NTRs have been observed in the inner ear and lateral line. BDNF has been found in the maculae of the utricle and saccule in the inner ear and the lateral line neuromast. NT-3 was localized to the sensory epithelium of the *crista ampullaris* of the utricle and saccule and to cells of the non-sensory epithelium lining the membranes of the membranous sacs. The expression of NGF was not identified within any part of the inner ear in these salmon species. Both TrkA and TrkB were present in small populations of sensory cells in the *crista ampullaris* and statoacoustic ganglion in both salmon species but absent in the sensory epithelium of the inner ear. Additionally, while TrkA and TrkB were widespread throughout the neuromasts in *S. salar*, in *S. trutta*, TrkA was confined to the mantle, and TrkB seemed restricted to lateral line hair cells. TrkC did not exhibit expression in the inner ear or lateral line (Germanà et al. 2002; Catania et al. 2007).

In the short-lived killifish (*N. guentheri*), the expression of TrkA and TrkC was detected in the semicircular canals, ciliary cells’ cytoplasm, and the apical part of the sensory patches of the utricle, saccule, and lagena of the inner ear. Moreover, the expression of both receptors was found in the central-apical part of the neuromasts, corresponding to the ciliated cells of the sideline. However, unlike in zebrafish (*D. rerio*), TrkB expression was absent in the mechanosensory system of this species (Aragona et al. 2021). For the lamprey (*L. fluviatilis*), a faint immunoreactive signal anti-NT-Lf was noted in the inner ear’s epithelial macula. Additionally, NTRs-like Lf-Trk1 and Lf-Trk2 were identified in the ganglionic structure between the macula and the otic foramen (Hallböök et al.^1998^).

The mechanosensory system of fish comprises a complex network of neural and non-neural cells, enabling them to detect and respond to mechanical stimuli. All the NTs and NTRs have been detected in this system of fish, mainly BDNF/TrkB, from early development until adulthood. However, their expression becomes increasingly restricted as the organism matures, from the most abundant expression in early stages to the expression in specialized cells in maturity. This trend is consistent across species, although the specific location of expression may vary. After injuries in neuromast or loss of hair cells, a regenerative process occurs to reform the missing part (Aragona et al 2022; Dufourcq 2006; Ghysen and Dambly‐Chaudière 2007; Williams and Holder 2000). If the mechanism to form and growth of the mechanosensory system are like those of regeneration (Thomas et al. 2015; Sánchez et al. 2016), such as BDNF/TrkB (Gasanov et al. 2015), the NT/NTR system should be similar in both processes, development, maintenance, and regeneration of mechanosensory system.

### Muscle and skin

The skin acts as a protective barrier against biological, chemical, and physical factors, possessing diverse functionalities such as thermoregulation and homeostasis, facilitating communication via visual cues, forming specific structures (scales, hair, and nails), enabling respiration, and serving as the largest sensory organ in the body with multiple sensory receptors (Proksch et al. 2008; Rakers et al. 2010; Talagas and Misery 2015). Both fish and mammalian skin share structural resemblances, characterized by a basement membrane that divides the epidermis and dermis. However, in fish, the epidermis is covered by epithelium rich in mucus (Hawkes 1974; Fast et al. 2002; Ángeles-Esteban 2012). In teleost fish, as in mammals, the body tissue consists of striated muscles. Yet, in fish, this muscle is packed into a series of blocks called myomeres, separated by a connective tissue sheet known as the myoseptum. These blocks exhibit a vertical “W-shaped” arrangement, enabling them to control lateral undulations and facilitate locomotion (Stiassny 2000).

The expression patterns of BDNF and TrkA, TrkB, and TrkC have been well documented in both muscle and skin tissues across vertebrates. NT-6/7, on the other hand, has been detected exclusively detected in fish. In the developmental stages of zebrafish (*D. rerio*), discrete points within the muscle layer of the pectoral fin bud exhibited BDNF expression (Hashimoto and Heinrich 1997). Additionally, adult zebrafish (*D. rerio*) showcased the presence of specific BDNF exons in both muscle and skin (Heinrich and Pagtakhan 2004; Blanco et al. 2020). In a salmon species (S. salar), IR anti-BDNF was observed in suprabasal epidermal cells (Germanà et al. 2002). Notably, NT-6 was first identified in the muscles and skin during organogenesis and later in mature tissues of swordtail fish (*X. maculatus*) (Götz et al. 1994). The skin of lampreys (*L. fluviatilis*) has been reported to express NT-Lf (Hallböök et al. 1998).

Regarding NTRs, cells expressing TrkA were located at the base of the pectoral fins of zebrafish (*D. rerio*) (Nittoli et al. 2018). Also, IR anti-TrkA was observed in chemosensory skin cells from seabass fish (*Dicentrarchus labrax*), in which TrkB was also detected in dendritic-like cells of the dermis (Hannestad et al. 2000). In salmon, IR anti-TrkB was detected in epidermal cells in *S. salar*, while anti-TrkC was observed in the head skin of *S. trutta* (Germanà et al. 2002).

It has only been possible to detect the presence of BDNF and NT-6/7 in developing and adult teleost fish and a little NT-Lf in lampreys. Conversely, the three high-affinity NTRs, TrkA, TrkB, and TrkC, have been identified in the skin or muscle across various species.

Among vertebrates, skin and muscle regeneration is similar, the growth factors that regulate the development and function of neurons are also involved in the control of skin homeostasis and remodeling (Rowlerson et al. 1997; Botchkarev et al. 2006; Nakatani et al. 2007; Seifert and Maden 2014). In fish, the dermis and skin derivatives can regenerate after an injury, without a recognizable scar remaining (Abe et al. 2019). The epidermis can synthesize NTs transport retrogradely to the ganglia and allow some cutaneous processes. Also, the NTs support the synthesis and development of sensory neurons that innervate the skin (Truzzi et al 2011).

Like the observed in the mechanosensory system, which shares a close morphological and functional relationship with the skin and muscles in fish, the presence of these molecules suggests that they participate in the development, maintenance, and regeneration of these tissues throughout the life of the organisms. Nevertheless, further research is needed to gain a better understanding of the specific roles played by NTs and NTRs in skin and muscle tissues characterized by a high regeneration rate in fish.

### Respiratory and circulatory system

Fish gills are complex tissues made up of epithelial and vascular surfaces, facilitating processes like respiration, osmoregulation, acid–base balance, metabolism of circulating hormones, and elimination of waste products of nitrogenous metabolism (Maetz 1971; Randall and Daxboeck 1984; Olson 1998). To enable respiration and gas exchange, fish propel water unidirectionally over the gills using muscles around the jaws and skeletal elements in the gill arches lining the pharynx. In contrast, other vertebrate groups, such as terrestrial animals, use a thoracic suction pump for breathing, achieving ventilation through coordinated contractions of various muscles (Taylor et al.^1999^).

In the typical cardiovascular system of fish, the heart propels blood to the gills, and from there, it is distributed to a systemic circuit (Taylor et al.^1999^). BDNF exons have been identified in the gills and heart of adult zebrafish (*D. rerio*) (Heinrich and Pagtakhan 2004; Blanco et al. 2020), along with their presence in cardiac progenitor cells (De Felice et al. 2014). Furthermore, the expression of NT-3 in the heart has been noted 24 and 48 h after fertilization (Nittoli et al. 2018). The fish-specific NT-6/7 has been detected in the gills and heart of swordtail fish (*X. maculatus*) during organogenesis and in adult tissues (Götz et al. 1994). Additionally, the exclusive NT for the lamprey (*L. fluviatilis*), NT-Lf, exhibits weak detection in the gills of this jawless fish (Hallböök et al. 1998). IR anti-TrkA was observed in the gill epithelium, TrkB in subepithelial dendritic-like cells of the gills, and TrkC in cells resembling pillar cells of sea bass (*D. labrax*) (Hannestad et al. 2000).

Fish gills and lungs have similarities in tissue structure, cell populations, genetic pathways, and some mechanisms of regeneration which are conserved across vertebrates. Both organs can be restored after an injury, requiring vascular and nerve supply, but the ability of fish to continuously grow and regenerate the gills throughout life is remarkable (Cadiz and Jonz 2020). Comparing respiratory organs can help to understand regeneration and the possible participation of NTs and NTRs in their mechanisms.

Contrary to what happens in mammals, the heart in most of the fish also can regenerate during their entire life (Gemberling et al. 2013; Sehring et al. 2016; Potts et al. 2021) generating opportunities to understand the cardiac system regeneration in vertebrates. In mammals, NTs and NTRs participate in angiogenesis smooth muscle control and cardiac myocytes (Caporali and Emanueli 2009). More studies are needed to compare the expression of NTs and NTRs in mammals and fish.

### Digestive system

Fish have adapted their digestive systems to fulfill the specific needs of each species, allowing them to consume a variety array of diets (Kramer and Bryant 1995; Buddington and Kuz’mina 2000a, b). The digestive system spans the entire body, primarily in the peritoneal cavity and enclosed by the mesentery. It consists of four main regions: the head, foregut, midgut, and hindgut (Buddington and Kuz’mina 2000a, b; Stevens and Hume 2004; Wilson and Castro 2010). The structure and functions of the digestive system remain uniform across species, encompassing processes such as digestion, osmoregulation, hormone secretion for metabolism and digestion, and protection against pathogens and harmful environmental components (Buddington and Kuz'mina 2000a, b; Wilson and Castro 2010).

Fish employ various strategies to enhance the surface area for digestion, such as elongating the gut, developing a complex, highly folded mucosa, using the pyloric fence (unique among vertebrates and composed of outpourings of the intestine), and featuring a spirally folded epithelium in the distal intestine. Additionally, essential components of the digestive system include the liver and pancreas (Buddington and Kuz'mina 2000a; Stevens and Hume 2004). The liver filters nutrients from the digestive tract, eliminating toxic compounds and producing bile that drains into the swim bladder or directly into the intestine. The pancreas secretes digestive enzymes, bicarbonates, and other ions into the intestine, along with endocrine secretions like insulin, glucagon, somatostatin, and pancreatic polypeptide (Buddington and Kuz'mina 2000b).

In zebrafish (*D. rerio*) larvae, NT-3 expression was detected in the pancreas, while NT-4/5 was detected in the intestinal bulb and midgut. Notably, the absence of detectable NTRs suggests a potential role for NTs in activating receptors through long-distance signals (Nittoli et al. 2018). In adults, BDNF expression was identified in the intestine and liver (Heinrich and Pagtakhan 2004). Furthermore, Montalbano et al. (2016) reported IR anti-BDNF and -TrkB in the gastrointestinal tract. Blanco et al. (2020) described the BDNF/TrkB-p75NTR system in various tissues; IR anti-BDNF was detected in the foregut and hindgut throughout the epithelium, in the lamina propria of the intestine. In the liver, anti-BDNF was seen in some scattered cells.

Lucini et al. (2003) observed IR anti-NGF and -NT-3 in the enteric nervous system of the stomach and intestine, as well as in the myenteric and submucosal plexus and epithelium of the small intestine in five teleost fish species: goldfish (*Carassius auratus*), sea bass (*Dicentrarchus labrax*), dorado (*Sparus aurata*), scorpionfish (*Scorpaena porcus*), and carp (*Hypophthalmichthys molitrix*); however, BDNF was not detected in this study. Additionally, the lamprey-specific NT, NT-Lf, was found in the liver (Hallböök et al. 1998).

All The NTRs were observed in the digestive system of fish. In zebrafish (*D. rerio*), TrkB and p75NTR were identified in the foregut and liver, but their presence in the hindgut was nearly undetectable (Blanco et al. 2020). De Girolamo et al. (1999) detected the IR anti-TrkB and -TrkC in gastric epithelial cells of three teleost fish species: sea bass (*D. labrax*), dorado (*S. aurata*), and scorpionfish (*S. porcus*). Abundant IR anti-TrkB was observed in the cecum sac and at the base of the stomach, where TrkC was also observed. However, IR anti-TrkA was not detected in the stomachs of these fish.

Conversely, Lucini et al. (1999) explored the presence of NTRs throughout the enteric nervous system and myenteric plexus in the same tree species and two others (goldfish (*C. auratus*) and carp (*H. molitrix*)). IR anti-TrkA was observed in several epithelial cells identified as endocrine outside the enteric nervous system. Meanwhile, IR anti-TrkB was observed within the surface and glandular epithelium of the stomach, while anti-TrkC was only found in the intestine of carp (*H. molitrix*) and the stomach of sea bass (*D. labrax*). In these five species of fish, TrkB and TrkC were observed to be present closely in the endocrine cells of the surface and glandular epithelium of the stomach.

Hannestad et al. (2000) observed IR anti-TrkA in sea bass (*D. labrax*) in cells of the oral duct and salivary glands, while endocrine cells and subepithelial dendritic-like cells were positive for TrkB, and endocrine cells and cells behind the epithelium were positive for TrkC. The pancreas showed IR anti-TrkA and -TrkB, although it is unknown which type of pancreatic cells expressed these molecules. In hybrid fish, the pantex (*Pagrus major* x *Dentex dentex*) and IR anti-TrkA, -TrkB, and -TrkC were detected in the enteric nervous system, mainly in the myenteric plexus. Furthermore, TrkB and TrkC were detected in intestinal epithelial endocrine cells (Radaelli et al. 2001).

The intestine can be considered one of the most primitive animal organs, and NTs and NTRs have been found in their digestive systems, regulating several digestive activities (Coulie et al. 2000; Chai et al 2003). The intestinal structure, development, and regeneration among vertebrates are very similar. Cellular process acts in normal renewal and regeneration after injury or diseases, intestinal cells differentiate to give rise to the new intestinal tissues and return to homeostasis following damage. During digestive system formation, cells get attached to a regenerative program and not only lose cell-type specificity but also acquire identity (Orlando et al. 2012; Lukonin et al. 2020). NTs and NTRs are involved in gut physiology and in the enteric nervous system of vertebrates (Luccini et al. 2002) suggesting they participate in develop, maintenance, and regeneration.

### Endocrine system

The endocrine system, in conjunction with the nervous system, is responsible for maintaining the homeostasis of vertebrate organisms and includes organs such as the pancreas, thyroid gland, and ovary, among others (Janz 2000).

In this system, only one NT has been identified, with the expression of NT-3 confirmed in the pancreas of zebrafish (*D. rerio*) (Nittoli et al. 2018), while IR for NTR were observed: anti-TrkA in the endocrine pancreas, -TrkB in both the endocrine and exocrine parenchyma, specifically in the peripheral cells of the pancreatic *acini* in scorpionfish (*S. porcus*) (Lucini et al. 2001), and -TrkC in the thyroid of sea bass (*D. labrax*) (Hannestad et al. 2000).

Ongoing research into the expression of various NTs and NTRs in these glands holds the potential to enhance our understanding of their roles in the body. Furthermore, it may lead to the development of novel treatments for disorders related to the endocrine system.

The pancreas shows a high degree of tissue regeneration (Joglekar et al 2007). Endocrine signals can affect regeneration by modulating immune response to injury, allocation of energetic resources, or by enhancing or inhibiting proliferation and differentiation pathways in regenerating tissue (Graf et al. 2006; Easterling et al. 2019). The presence of NTs and NTRs in the endocrine system suggests their involvement in the development, maintenance, and regeneration of vertebrates.

### Urinary system

The structure of the fish urinary system undergoes significant variation with respect to terrestrial vertebrates, based on the habitat of each species, grouping them broadly into saltwater, brackish water, and freshwater fish. These structural modifications also hinge on the body type of each species, including elongated, compressed, or intermediate forms, and there are also differences between sexes. The well-developed bladder is accompanied by a short urethral duct that concludes in the urogenital papilla located behind the anus (Hentchel and Elger 1987; de Girolamo et al. 2000; Hentchel et al. 2000).

For instance, in freshwater goldfish (*C. auratus*), cells displaying immunoreactive anti-NGF and -NT-3 were detected along arteries adjacent to afferent arterioles. In marine scorpionfish (*S. porcus*), these cells were observed in afferent arterioles and adjacent secondaries. Both species exhibited co-expression of NGF and NT-3 in kidney JG cells (Arcamone et al. 2005).

IR anti-NTRs were detected in these two species and the seabass fish (*D. labrax*), in tubule cells and the kidney’s collecting ducts. IR anti-TrkA was observed in the kidney of all three species in numerous cells in the collecting duct system, while TrkC was only observed in goldfish (*C. auratus*) in the distal collecting duct system of the epithelium (de Girolamo et al. 2000,2003). IR anti-TrkA and -TrkB were confirmed in sea bass (*D. labrax*) in the long tubules of the head-of the kidney, while IR anti-TrkC was observed in the luminal pole of tubular epithelial cells and the hematopoietic parenchyma (Hannestad et al. 2000).

Between zebrafish (*D. rerio*) and mammalian kidneys, there exist several similarities; both have nephrons with a glomerulus, proximal tubules, distal tubules, and collecting ducts. In this fish species, pronephric tubules exhibited IR anti-TrkB and -p75NTR (Cacialli et al. 2018), and their kidneys express NGF, BDNF, NT-3, TrkA, TrkB, and TrkC, being the most abundant NT-3 and TrkC (Cacialli and Lucini 2022). The presence of these NTs and NTRs in the kidneys of both freshwater and marine fish implies their involvement in various cellular processes, including the mediation of growth, differentiation, survival, and repair of tubule cells (Arcamone et al. 2005).

Regeneration of the kidney is conserved in vertebrate evolution; on the contrary, nephron regeneration is high in fishes. In mammals, the regenerative capacity of the kidney does not lead to the generation of new nephrons as occurs in fish, but the three main strategies of response to kidney injury (cellular regeneration, nephron neogenesis, and kidney hypertrophy) have existed since the appearance of our fish ancestors (Romagnani et al. 2013; Davidson 2014; Bates et al. 2018). Understanding the mechanisms that drive renal progenitor activation, growth, and differentiation contributes to a better understanding of their development, maintenance, and regenerative mechanisms including the NTs and NTRs participation.

### Reproductive system

The vertebrate reproductive system comprises gonads, responsible for gamete and sex hormone production, gonoducts or genital ducts for gamete transport, and, in certain instances, external genitalia. Among vertebrates, fish showcase remarkable diversity in reproductive strategies and anatomical adaptation tailored to optimize fertilization in varied environments. Most species are gonochoric and sexually dimorphic, exhibiting distinct morphologies between males and females. However, some species may manifest as synchronous hermaphrodites, expressing both male and female traits simultaneously, or as sequential hermaphrodites, altering their sex at specific life stages. Moreover, there are asexual gynogenic species that inherit only the maternal genome (Shapiro et al. 1984; Redding and Patiño 2000; Penman and Piferrer 2008).

Gamete production in fish can occur synchronous and unitary within sexual partners, in groups with synchronous release into the environment at specific times, or asynchronous, involvement repeated gamete production and spawning over prolonged periods (Redding and Patiño 2000). While most species utilize external fertilization with oviparous embryonic development, some opt for internal fertilization and are typically viviparous (Worms and Lombardi 1992).

NTs play an essential role in regulating intracellular communication within organs involved in gametogenesis. With the exception of NT-6/7 and their corresponding NTRs, all NTs have been identified during the fetal development of testicles and in the adult tissue of some vertebrates, as well as ovarian follicles, granulosa cells, and the ovary (Ojeda et al. 2000; Jensen and Johnson 2001; Seifer et al. 2002,2006; Paredes et al. 2004; Kawamura et al. 2005; Harel et al. 2006; Müller et al. 2006; Guerra et al. 2013; Li and Zhou 2013; Streiter et al. 2016; Cacialli et al. 2018).

Transcript profiles of BDNF in testicular cells of zebrafish (*D. rerio*) and sperm of goldfish (*C. auratus*) were analyzed, revealing higher BDNF transcripts in fish with good-quality sperm, indicating its potential as a molecular marker for sperm quality (Guerra et al. 2013). In zebrafish (*D. rerio*) gonads, both male and female, low levels of BDNF, TrkB, and p75NTR were detected (Cacialli et al. 2018; Blanco et al. 2020). IR anti-BDNF was detected in type B spermatogonia, the apical part of the spermatid cytoplasm, and Sertoli cells, with a weaker signal in type A spermatogonia. In A and B spermatogonia and dyplotenic spermatocytes, NGF and TrkA mRNA expression were detected (Cacialli 2022).

In the ovary, BDNF signal intensity decreased as oocytes matured, with the strongest signal observed in the perinuclear cytoplasm of stage I oocytes and in the follicular layer cells surrounding the oocyte. BDNF likely plays a role in regulating oocyte growth and maturation of primary oocytes. No signals for TrkB were detected in the tests or ovary, and p75NTR was only detected in stage III follicular layer cells, suggesting its involvement in regulating folliculogenesis (Cacialli et al. 2018).

NTs actions happen in gonads in females and play important roles in the maturation of follicles and ovulation by promoting the initial differentiation and the subsequent growth of primordial follicles (Dissen et al. 202). In males, the development of the testes and their germ cells is governed by several cellular processes with an expression of NTs and NTRs (Li and Zou 2013. The presence of NTs and NTRs in fish gonads suggests their participation in the development, maintenance, and regeneration of follicles and tests of fish.

### Immune system

In teleost fish, the primary defense line includes the skin, lateral line, and gills, while the thymus produces T cells to enhance macrophage function and stimulate B cells. The spleen, acting as an antigen, traps and facilitates lymphocyte stimulation and proliferation (Romano et al. 1997; Warr 1997; Powell 2000). The detection of NTs and NTRs in fish’s immune system suggests their participation in immune cell development, function, and communication.

Among fish, two NTs have been identified in this system, the fish-specific NT-6, detected during organogenesis and in adult spleen tissues of swordtail fish (*X. maculatus*) (Götz et al. 1994), and BDNF, found in low levels in the spleen of zebrafish (*D. rerio*), indicating the presence of this NT in the fish immune system (Blanco et al. 2020). On the other hand, all NTRs have been identified in the immune system of fish. Immunoreactive anti-TrkA was detected in a small group of thymic cells, anti-TrkB in scattered dendritic-like cells, and anti-TrkC in parenchymal cells below the epithelial lining, and in cells near the septum of the spleen (Hannestad et al. 2000). Further research in this area will deepen our understanding of the complex interactions between NTs and the immune system in fish, potentially leading to novel insights into immune-related diseases and the development of targeted therapeutic approaches.

Sentence added: “Immune system participate for tissue regeneration by enhancing or inhibiting proliferation and differentiation pathways” (Graf et al. 2006; Easterling et al. 2019).

### Conclusions and perspectives

Neurotrophins (NTs) and their receptors (NTRs) affect the development, maintenance, and regeneration of diverse cell populations, albeit they are associated commonly with actions in neurons including dendritic growth (Bibel and Barde 2000) regulating neuronal connectivity in youth and adulthood (Lewin and Barde 1996). Nevertheless, these molecules have been identified beyond the nervous system (e.g., Hoener et al. 1996; Shibayama and Koizumi 1996; Hannestad et al. 1998; Lommatzsch et al.^1999^; Lucini et al. 1999, 2001, 2002) indicating that also they can act on non- neural tissue regulating the survival, anti-inflammation, proliferation, and differentiation (Xiao and Le 2016).

As reviewed above, the BDNF was the NT most frequently identified outside the CNS of teleost fish, closely followed by NT-3, underscoring their widespread utilization and popularity in laboratory procedures, highlighting their significance in both neural and NNT. The pairs BDNF/TrkB and NT-3/TrkC were notably present in the PNS of the heavily innervated mechanosensory system. Indeed, a high frequency of BDNF and NT-3 expressions was found in the digestive system. On the contrary, BDNF was absent in the endocrine and urinary systems of fish. Similarly, NT-3 was not detected in the muscle, skin, reproductive, and immune systems of fish, despite being found in these systems in other vertebrates. This underscores the need to explore and understand their roles in these tissues/organs/systems.

NGF has been exclusively observed in the mechanosensory, digestive, and urinary systems. Nonetheless, its high-affinity receptor, TrkA, was found in almost all tissues/organs/systems, suggesting potential functional roles for NGF in those tissues. In various vertebrates, the NGF/TrkA/p75NTR complex is recognized for its involvement in cell proliferation and regeneration across diverse tissues/organs/systems. Therefore, it is plausible to expect that the NT/NTR complex may perform similar functions in fish.

The exploration of fish-exclusive NTs has been constrained; however, they have been identified both within and outside the CNS. NT-6/7, acknowledged as an NGF homolog, may function similarly. The mechanisms underlying their expression represent an expanding opportunity for further research.

The expression of TrkA, TrkB, and TrkC ascertained in different tissues likely implies actions of NTs still unreported. Among analyzed tissues, the reproductive one seemingly expresses TrkB alone. The low-affinity receptor (p75NTR) has been identified in the digestive, urinary, and reproductive systems, which are interconnected.

The expression and localization of NTs and NTRs vary among organs, with some exhibiting more significant expression than others. All of them were detected in the fish mechanosensory system, given its association with the peripheral nervous system (PNS). However, in other organs, their expression differs in significance.

Significantly, the digestive system distinguishes itself, with nearly all NTs, except for NT-6/7, identified in 15 cited articles. Such a ubiquitous pattern is associated with the complexity of the myenteric nervous system, which regulates various gastrointestinal functions. For other vertebrates, especially in mammals, the detection of NTs and NTRs has revealed their influence on gut physiology like sensation, motility, epithelial barrier integrity, neuroprotection, and neuroplasticity, occurring both during development and in the adult organism (Liu 2018). In fish, similar functions may be attributed to the expression of NTs and NTRs. Certainly, aquatic lifestyle adaptations of fish exhibits may vary among different groups of vertebrates, and NTs contributions should be further addressed with a focus on appetite stimulation, lipid and glucose metabolism regulation, and indirect regulation of food intake.

With the exception of NGF and NT-4/5, NTs and NTRs have been detected in fish respiratory and circulatory systems. At the CNS level, regulatory mechanisms of respiratory and heart rates of lower chordates, such as fish, exhibit a remarkable degree of homology with those operating in mammals (Tylor 1989), albeit there are some differences that may be associated with NTs specialized actions (e.g., bronchi function and NGF expression in mammals (Liu et al. 2021)). Cardiac cells are targets for NTs from early development through maturity, contributing to cardiovascular homeostasis. Specifically, BDNF and NT-3 are crucial for the well-developed cardiac system in mammals (Emanueli et al 2014; Pius-Sadowska and Machalinski 2017), suggesting potential implications in fish systems as well.

The detection of all three high-affinity NTRs in fish suggests the potential involvement of other NTs in the endocrine system. Additionally, all NTRs have been reported in pituitary endocrine cells (Taglialatela et al. 1991; Shibayama and Koizumi 1996; Aguado et al. 1998). Fish NT-3 may function similarly to mammalian NGF; however, its elucidation remains to be approached.

Both NGF and NT-3 have been observed in the kidneys and afferent ducts of both freshwater and saltwater fish, suggesting a conserved role for these neurotrophins across diverse aquatic environments. Moreover, the detection of TrkA, TrkB, and TrkC receptors in various kidney structures such as tubules and collecting ducts highlights the widespread distribution of neurotrophin receptors in fish species. Notably, the identification of p75NTR in pronephric tubules underscores the diverse localization of neurotrophin receptors within the fish kidney. These findings underscore the intricate interplay between the nervous system and renal function, implicating neurotrophins in crucial cellular processes such as growth, differentiation, survival, and repair of tubule and kidney cells (Arcamone et al. 2005). Furthermore, their presence suggests a potential role in mediating species-specific adaptations and physiological functions of the urinary system. In mammals, it is well documented that neurotrophins like NGF and BDNF play pivotal roles in both normal urinary function and pathological conditions such as incontinence (Ochodnicky et al. 2012; Song et al. 2014). As such, ongoing research into the expression patterns and functional significance of neurotrophins and their receptors in the urinary system of vertebrates, particularly in fish, holds promise for advancing our understanding of kidney biology. Moreover, these studies may pave the way for the development of novel therapeutic interventions targeting urinary system disorders.

Notably, BDNF/TrkB has been identified in the reproductive system of fish that extends beyond the gonads to sperm and ova, implying a crucial involvement in their developmental and successful reproduction. In males, BDNF has been observed in spermatogonia, spermatids, and Sertoli cells and is linked to sperm quality (Guerra et al. 2013; Cacialli et al. 2018; Blanco et al. 2020). In females, BDNF has been detected in the gonads (Cacialli et al. 2018; Blanco et al. 2020). In mammals, all NTs and NTRs have been detected in the ovaries, and tests underscore their importance in reproductive development. For example, BDNF/TrkB has been detected before the primordial follicle growth, NGF (Dissen et al. 2009; Chow et al. 2020), NGF and NT-3 in Sertoli cells (Cupp et al. 2000; Perrard et al 2007; Li and Zhou 2013), suggesting their involvement in ovary growth and maturation, and spermatogenesis across vertebrates. Despite these findings, the precise mechanisms underlying the actions of NTs and NTRs in the reproductive system remain incompletely understood. Understanding the expression patterns and functions of NTs and NTRs in fish can contribute to our knowledge of reproductive biology across all vertebrates.

The immune system comprises a complex network of specialized cells, tissues, and organs that defend against pathogens (Kisia 2016). In fish, BDNF and NT-6/7, alongside all three high-affinity NTRs, have been identified in the spleen and thymus from early development to adulthood. In mammals, NTs and NTRs demonstrate diverse functions within the immune system, spanning from early development to adulthood and even during illness (Lomen-Hoerth and Shooter 1995; Otten and Gadient 1995; Kerschensteiner et al. 2003; Linker et al. 2009). This intricate involvement highlights the dynamic interaction between the nervous and immune systems, influencing both normal physiological processes and pathological conditions.

The studies considered here primarily focused on investigating the localization and expression of neurotrophins and their receptors, either as genes or as proteins. However, their exact roles in biological processes remain poorly understood. During the early stages of fish development, NTs and NTRs are expressed more abundantly than in adult life, suggesting their significant contribution throughout development. Nevertheless, these molecules also continue to be expressed in adult life, indicating their involvement in the maintenance and regeneration of tissues.

The localization and identification of NTs and NTRs beyond the CNS have been documented in mammals since their discovery. NTs expressions are observed across various non-neuronal tissues, where it promotes tissue regeneration, inhibits apoptosis, and enhances proliferation and functional recovery. In some cases, NTs levels increase following injury, suggesting a role in tissue repair mechanisms (Xiao and Le 2016). NTs are expressed in hematopoietic stem cells, where they enhance proliferation and vascularization. It is also found in cardiac myocytes and myocardium, salivary gland ducts, and pancreas. In testis and epididymis, it facilitates germ cell differentiation. In bone marrow stem cells, NTs increase the colony-forming unit of granulocytes and monocytes, promote differentiation into B lymphocytes or neuron-like cells, and enhance their survival. Remarkably, NTs can also stimulate cell proliferation in certain cancer cell types. NTRs are similarly significant in various tissues and processes, including the basal and granular layers of the epidermis, cardiac tissue, digestive system, and kidney, which promotes nephrogenesis, pancreas, prostatic epithelial cells, ovarian theca and granulosa cells, endometrium, mammary ducts, in spermatogenesis, hair follicle cycle regulation, spleen, and immune cells such as lymphocytes, monocytes, and mast cells. Despite these findings, the mechanisms underlying the roles of NTs and NTRs remain to be fully elucidated (see Shibayama et al., 1996; Sariola 2001; Xiao and Le 2016).

In addition, the present review highlights the decline in research on fish NNT in recent years, emphasizing the need for renewed efforts to explore NTs and NTRs in non-conventional species. Increasing the knowledge about the localization of their expression out of CNS could allow a better understanding of their functions and how they can participate in the differentiation, migration, growth, survival, regeneration, and death of distinct cell populations. Further research in these areas will deepen our understanding of fish physiology, provide insights into metabolic and reproductive disorders, and potentially yield novel therapeutic interventions for diseases related to tissues/organs/systems. Analyzing both NTs and NTRs expression in teleost fish can contribute to a broader comprehension of these molecules in more complex vertebrates, including humans. It holds the potential to advance our understanding of tissue development, maintenance, and regeneration, as well as to uncover new avenues for addressing diseases related to tissues/organs/systems.

## Data Availability

No datasets were generated or analysed during the current study.

## Declarations

### Statements and declarations

The authors confirm that the manuscript has not been published before and has been read and approved.
